# Using permeation guidelines to design new antibiotics—A PASsagE into *Pseudomonas aeruginosa*

**DOI:** 10.1002/ctm2.1600

**Published:** 2024-03-01

**Authors:** Brett N. Cain, Paul J. Hergenrother

**Affiliations:** ^1^ Department of Chemistry and Carl R. Woese Institute for Genomic Biology University of Illinois Urbana Illinois USA

Dear Editor,

The ability to employ small molecules to kill pathogenic bacteria and prevent serious illness or death has been one of mankind's greatest advances, as before the advent of the first antibiotics (salvarsan, penicillin) approximately 30% of deaths in the United States and United Kingdom were due to bacterial infections.^1^ This shield against disease, however, is weakening as resistance to approved antibiotics rises. Worryingly, no new class of antibiotics with activity against gram‐negative bacteria has been approved by the FDA in over 55 years,^2^ with *Pseudomonas aeruginosa* being of particular concern. Multidrug‐resistant *P. aeruginosa* is classified as a “serious threat” by the CDC and there are only four antibiotic classes regularly used to treat *P. aeruginosa* infections (β‐lactams, aminoglycosides, fluoroquinolones, and polymyxins).^3^, ^4^ This dearth of therapeutic options makes the occurrence of drug‐resistant *P. aeruginosa* even more troubling, and three of these drug classes (aminoglycosides, fluoroquinolones, and polymyxins) have serious toxicity concerns. As it stands approximately 85 000 deaths globally were attributed to antibiotic‐resistant *P. aeruginosa* in 2019 alone, placing it among the six most deadly pathogens.^5^ Gram‐negative pathogens are notorious for being difficult to penetrate with small molecules, and *P. aeruginosa* is particularly problematic in this regard, as it does not possess general diffusion porins and has many general and promiscuous efflux pumps.^6^, ^7^ Accordingly, a primary challenge in the development of new therapeutics for *P. aeruginosa* is achieving effective drug concentrations inside the cell, where many valuable antibacterial targets exist.^8^ To address this challenge, we have recently disclosed general permeation guidelines for *P. aeruginosa*, facilitating the design of small molecules that hijack the bacterium's self‐promoted uptake pathway and gain intracellular access.^9^

For this work, the intracellular accumulation of individual members from a large and diverse compound collection was determined using an LC–MS/MS assay.^10^ Testing a diverse array of small molecules was critical for success, ensuring the results were not limited to compound classes possessing antibacterial activity. In addition to more typical drug‐like small molecules, the collection contained novel, natural‐product‐like compounds constructed via the “complexity‐to‐diversity” approach.^11^ After experimentally measuring individual accumulation values for 345 compounds, the resulting data were interpreted with the help of a random forest classification model that pointed to certain characteristics associated with positive charge and hydrogen bond donation as important for *P. aeruginosa* accumulation. Specifically, compounds with appropriate positive polar surface area (Q_VSA_PPOS ≥ 80) and/or a positive formal charge (FC ≥ .98), as well as sufficient hydrogen bond donor surface area (HBDSA ≥ 23) have a high likelihood of accumulating in *P. aeruginosa*.^9^

The general permeation guidelines for *P. aeruginosa* can now be compared and contrasted with permeation guidelines disclosed in 2017 for another gram‐negative pathogen, *Escherichia coli*.^12^ While there are similarities, the differences in the described physicochemical characteristics are linked back to differences in bacterial physiology (Figure 1). The guidelines pertaining to *E. coli*—the eNTRy rules^12^, ^13^—are rooted in optimizing small molecule entry through its major water‐filled transport proteins called porins. In brief, the 2017 study found that the presence of an ionizable nitrogen on a compound with low three‐dimensionality and high rigidity enabled accumulation in *E. coli*.^12^ The spatial characteristics of rigidity and globularity are important for traversing the narrow porin channel constriction zone, which ranges from approximately 7 to 11 angstroms.^14^ The critical presence of an ionizable nitrogen has been traced to its ability to engage in favourable electrostatic interactions with negatively‐charged amino acids that line the porin constriction zone.^12^, ^15^, ^16^, ^17^ The *P. aeruginosa* permeation guidelines^9^—dubbed here the PASsagE rules (***P** seudomonas **a** eruginosa* **S** elf‐promoted **E** ntry)—in contrast provide guidance for tailoring small molecules to productively access the self‐promoted uptake pathway for entry into the cell, consistent with recent work from the Bumann lab suggesting that porins are not important for antibiotic uptake in *P. aeruginosa*.^18^ Notably, and in contrast with the eNTRy rules, the PASsagE rules have no flexibility and shape requirements, and instead appear to optimize favourable interactions of the small molecule with the gram‐negative outer membrane through sufficient positive polar charge and HBDSA.

**FIGURE 1:**
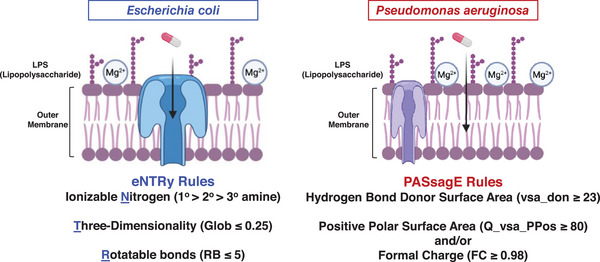
Differences in outer membrane physiology and permeation guidelines for gram‐negative bacteria *Escherichia coli* and *Pseudomonas aeruginosa*, with eNTRy‐rule compliant compounds able to enter *E. coli* through porins, and PASsagE (*Pseudomonas aeruginosa* Self‐promoted Entry)‐compliant compounds entering via the self‐promoted uptake pathway. *Source*: Parameters taken from Richter et al.^12^ and Geddes et al.^9^ ^.^

Antibacterial drug discovery requires the developing team to balance a range of attributes including target essentiality, homology with analogous human target(s), toxicity, and druggability.^19^ For projects centred around combatting gram‐negative bacteria, cellular permeability is also a particularly salient attribute. Often, there is a discordance between target engagement and access to the target, which manifests in reduced activity in whole‐cell assays. For example, many gram‐positive antibiotics are in fact effective against *P. aeruginosa* when tested against genetically engineered permeability‐defect strains.^20^ To this end, these new rules can provide guidance to development teams about what modifications to make to enhance the accumulation of candidate antibiotics analogous to the routine procedure of optimizing lead compounds for solubility and pharmacokinetic properties based on known, fundamental principles. For a typical small molecule lead there are moieties that are critical for target binding (pharmacophore), and tangential functionality (auxophore) that can be altered in the optimization of non‐target related properties, such as toxicity or solubility.^21^ These newly disclosed permeation rules can now be used to guide auxophore modification for enhanced *P. aeruginosa* accumulation. The eNTRy rules, put forth in 2017, have already accelerated gram‐negative antibacterial campaigns and delivered high accumulating and active antibiotics via modification of the auxophore in many different contexts.^22^, ^23^, ^24^, ^25^, ^26^, ^27^, ^28^, ^29^, ^30^, ^31^, ^32^, ^33^

Often, the eNTRy rules are used in campaigns that are centred around a small molecule that already possesses the necessary spatial parameters but lacks the amine required for gram‐negative uptake. Although other ionizable nitrogen moieties can be beneficial for *E. coli* accumulation,^34^ primary amines typically result in the largest accumulation increase and are thus typically preferred. While the PASsagE rules do require positive polar charge to be present, this can take many forms beyond a primary amine. This can be observed, retrospectively, by considering tetracyclines. Although tetracycline itself has activity against many prominent gram‐negative pathogens, including *E. coli*, it has an elevated minimum inhibitory concentration against *P. aeruginosa*, partially explained by the differential accumulation of tetracycline in these two pathogens (Figure 2A).^9^ However, additions of a secondary amine, tertiary amine, and secondary amide produce tigecycline, a compound within the PASsagE rules and one that gains accumulation and whole cell antibiotic activity against *P. aeruginosa* (Figure 2A). Tetracycline and tigecycline have nearly equivalent activity against a permeability‐defect strains of *P. aeruginosa*,^9^ suggesting that target engagement is similar between these two compounds; efflux could also play a role in the differential activity of these compounds.^35^

**FIGURE 2:**
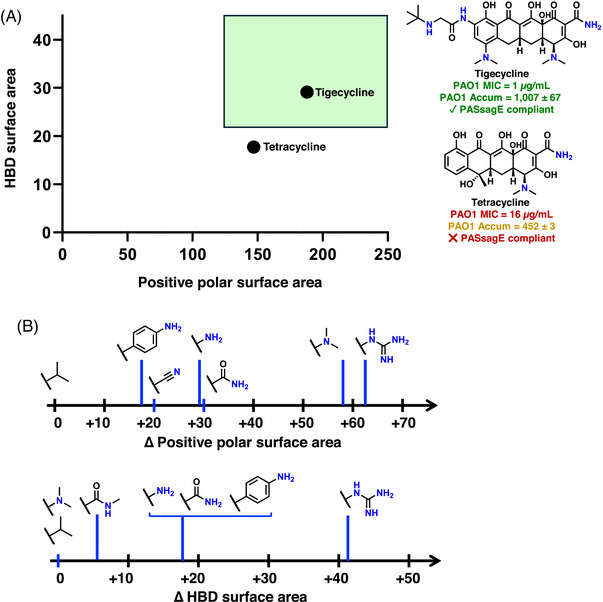
(A) A retrospective consideration of the conversion of tetracycline to tigecycline, a version more active against wild‐type *Pseudomonas aeruginosa*. Notably, these two compounds have similar target engagement as judged by nearly identical minimum inhibitory concentration (MIC) values against an efflux‐deficient strain of *P. aeruginosa* PAO1 (0.25 µg/mL and 0.125 µg/mL respectively); accumulation values are in units of nmol/10^12^ CFUs.^9^ The green box represents the PASsagE‐compliant region. (B) The value of certain nitrogenous functional groups in enhancing physiochemical parameters important for *P. aeruginosa* uptake. Values calculated using cyclohexane with the represented functional groups appended, using molecular operating environment (MOE) after assessing the compounds protonation state at physiological pH. HBD, hydrogen bond donor.

The liberation from shape and flexibility requirements, and the unmooring from a primary amine, offers the medicinal chemist considerably more freedom in the chemical moieties available to tailor a small molecule to become PASsagE‐compliant. Assessment of a generic scaffold shows the variety of functional groups able to increase HBDSA and polar positive surface area (Figure 2B), with some moieties (e.g., guanidiniums) altering both parameters simultaneously. With no restriction on spatial parameters, and no need for a primary amine, there is significant flexibility in the types of functionalities that can be added to a compound and aid in *P. aeruginosa* accumulation.

The futility of whole‐cell screening of industrial‐style compound collections against gram‐negative bacteria has been well‐documented.^36^, ^37^, ^38^ The reasons behind this are not a mystery, as the large compound screening collections used by most research groups (academia and pharmaceutical industry) lack members with traits enabling gram‐negative cell penetration; for example, only .1% of compounds in the Chembridge Microformat Library possess a primary amine.^39^ As compounds predisposed to penetrate gram‐negative bacteria are not present in standard screening collections, turning to other sources for novel chemical matter makes sense with recent successes in discovering interesting antibacterial leads through mining “unculturable” bacteria,^40^ examining natural products produced by eukaryotic symbionts,^41^ and using non‐standard chemical matter.^42^ A complimentary approach is chemically modifying a non‐permeable antibiotic with functional groups that enhance accumulation; accumulation rules will guide such modifications and facilitate development of potent antibiotics.

## FUNDING INFORMATION

National Institutes of Health, Grant/Award Number: AI176523.

## References

[1] Bergkessel M , Forte B , Gilbert IH . Small‐molecule antibiotic drug development: need and challenges. ACS Infect Dis. 2023;9(11):2062‐2071. doi:10.1021/acsinfecdis.3c00189 37819866 PMC10644355

[2] Lewis K . Platforms for antibiotic discovery. Nat Rev Drug Discov. 2013;12(5):371‐387. doi:10.1038/nrd3975 23629505

[3] CDC . 2019 Antibiotic Resistance Threats Report . Accessed January 4, 2024. https://www.cdc.gov/drugresistance/pdf/threats‐report/2019‐ar‐threats‐report‐508.pdf

[4] Driscoll JA , Brody SL , Kollef MH . The epidemiology, pathogenesis and treatment of *Pseudomonas aeruginosa* infections. Drugs. 2007;67(3):351‐368. doi:10.2165/00003495-200767030-00003 17335295

[5] Antimicrobial Resistance Collaborators . Global burden of bacterial antimicrobial resistance in 2019: a systematic analysis. Lancet. 2022;399(10325):629‐655. doi:10.1016/S0140-6736(21)02724-0 35065702 PMC8841637

[6] Chevalier S , Bouffartigues E , Bodilis J , et al. Structure, function and regulation of *Pseudomonas aeruginosa* porins. FEMS Microbiol Rev. 2017;41(5):698‐722. doi:10.1093/femsre/fux020 28981745

[7] Aeschlimann JR . The role of multidrug efflux pumps in the antibiotic resistance of *Pseudomonas aeruginosa* and other gram‐negative bacteria. Insights from the Society of Infectious Diseases Pharmacists. Pharmacotherapy. 2003;23(7):916‐924. doi:10.1592/phco.23.7.916.32722 12885104

[8] Yoshimura F , Nikaido H . Permeability of *Pseudomonas aeruginosa* outer membrane to hydrophilic solutes. J Bacteriol. 1982;152(2):636‐642. doi:10.1128/jb.152.2.636-642.1982 6813310 PMC221510

[9] Geddes EJ , Gugger MK , Garcia A , et al. Porin‐independent accumulation in *Pseudomonas* enables antibiotic discovery. Nature. 2023;624(7990):145‐153. doi:10.1038/s41586-023-06760-8 37993720 PMC11254092

[10] Geddes EJ , Li Z , Hergenrother PJ . An LC‐MS/MS assay and complementary web‐based tool to quantify and predict compound accumulation in *E. coli* . Nat Protoc. 2021;16(10):4833‐4854. doi:10.1038/s41596-021-00598-y 34480129 PMC8715754

[11] Huigens RW , Morrison KC , Hicklin RW , Flood TA , Richter MF , Hergenrother PJ . A ring‐distortion strategy to construct stereochemically complex and structurally diverse compounds from natural products. Nat Chem. 2013;5(3):195‐202. doi:10.1038/nchem.1549 23422561 PMC3965367

[12] Richter MF , Drown BS , Riley AP , et al. Predictive compound accumulation rules yield a broad‐spectrum antibiotic. Nature. 2017;545(7654):299‐304. doi:10.1038/nature22308 28489819 PMC5737020

[13] Richter MF , Hergenrother PJ . The challenge of converting gram‐positive‐only compounds into broad‐spectrum antibiotics. Ann NY Acad Sci. 2019;1435(1):18‐38. doi:10.1111/nyas.13598 29446459 PMC6093809

[14] Cowan SW , Schirmer T , Rummel G , et al. Crystal structures explain functional properties of two *E. coli* porins. Nature. 1992;358(6389):727‐733. doi:10.1038/358727a0 1380671

[15] Haloi N , Vasan AK , Geddes EJ , et al. Rationalizing the generation of broad spectrum antibiotics with the addition of a positive charge. Chem Sci. 2021;12(45):15028‐15044. doi:10.1039/d1sc04445a 34909143 PMC8612397

[16] Acharya A , Jana K , Kleinekathofer U . Antibiotic charge profile determines the extent of L3 dynamics in OmpF: an expedited passage for molecules with a positive charge. J Phys Chem B. 2023;127(50):10766‐10777. doi:10.1021/acs.jpcb.3c04557 38064341

[17] Maher C , Maharjan R , Sullivan G , Cain AK , Hassan KA . Breaching the barrier: genome‐wide investigation into the role of a primary amine in promoting *E. coli* outer‐membrane passage and growth inhibition by ampicillin. Microbiol Spectr. 2022; 10(6):e0359322. doi:10.1128/spectrum.03593-22 36409154 PMC9769794

[18] Ude J , Tripathi V , Buyck JM , et al. Outer membrane permeability: antimicrobials and diverse nutrients bypass porins in *Pseudomonas aeruginosa* . Proc Natl Acad Sci USA. 2021;118(31):e2107644118. doi:10.1073/pnas.2107644118 34326266 PMC8346889

[19] Theuretzbacher U , Blasco B , Duffey M , Piddock LJV . Unrealized targets in the discovery of antibiotics for gram‐negative bacterial infections. Nat Rev Drug Discov. 2023;22(12):957‐975. doi:10.1038/s41573-023-00791-6 37833553

[20] Krishnamoorthy G , Leus IV , Weeks JW , Wolloscheck D , Rybenkov VV , Zgurskaya HI . Synergy between active efflux and outer membrane diffusion defines rules of antibiotic permeation into gram‐negative bacteria. mBio. 2017;8(5):e01172‐17 doi:10.1128/mBio.01172-17 PMC5666154 29089426

[21] Stoorza AM , Duerfeldt AS . Guiding the way: traditional medicinal chemistry inspiration for rational gram‐negative drug design. J Med Chem. 2024;67(1):65‐80. doi:10.1021/acs.jmedchem.3c01831 38134355 PMC11342810

[22] Motika SE , Ulrich RJ , Geddes EJ , Lee HY , Lau GW , Hergenrother PJ . Gram‐negative antibiotic active through inhibition of an essential riboswitch. J Am Chem Soc. 2020;142(24):10856‐10862. doi:10.1021/jacs.0c04427 32432858 PMC7405991

[23] Parker EN , Cain BN , Hajian B , et al. An iterative approach guides discovery of the FabI inhibitor fabimycin, a late‐stage antibiotic candidate with in vivo efficacy against drug‐resistant gram‐negative infections. ACS Cent Sci. 2022;8(8):1145‐1158. doi:10.1021/acscentsci.2c00598 36032774 PMC9413440

[24] Parker EN , Drown BS , Geddes EJ , et al. Implementation of permeation rules leads to a FabI inhibitor with activity against gram‐negative pathogens. Nat Microbiol. 2020;5(1):67‐75. doi:10.1038/s41564-019-0604-5 31740764 PMC6953607

[25] Hu Y , Shi H , Zhou M , et al. Discovery of pyrido[2,3‐b]indole derivatives with gram‐negative activity targeting both DNA gyrase and topoisomerase IV. J Med Chem. 2020;63(17):9623‐9649. doi:10.1021/acs.jmedchem.0c00768 32787097

[26] Andrews LD , Kane TR , Dozzo P , et al. Optimization and mechanistic characterization of pyridopyrimidine inhibitors of bacterial biotin carboxylase. J Med Chem. 2019;62(16):7489‐7505. doi:10.1021/acs.jmedchem.9b00625 31306011 PMC6980355

[27] Lukezic T , Fayad AA , Bader C , et al. Engineering atypical tetracycline formation in amycolatopsis sulphurea for the production of modified chelocardin antibiotics. ACS Chem Biol. 2019;14(3):468‐477. doi:10.1021/acschembio.8b01125 30747520

[28] Skepper CK , Armstrong D , Balibar CJ , et al. Topoisomerase inhibitors addressing fluoroquinolone resistance in gram‐negative bacteria. J Med Chem. 2020;63(14):7773‐7816. doi:10.1021/acs.jmedchem.0c00347 32634310

[29] Brem J , Panduwawala T , Hansen JU , et al. Imitation of beta‐lactam binding enables broad‐spectrum metallo‐beta‐lactamase inhibitors. Nat Chem. 2022;14(1):15‐24. doi:10.1038/s41557-021-00831-x 34903857

[30] Schumacher CE , Rausch M , Greven T , Neudorfl JM , Schneider T , Schmalz HG . Total synthesis and antibiotic properties of amino‐functionalized aromatic terpenoids related to erogorgiaene and the pseudopterosins. Eur J Org Chem. 2022;2022(26):e202200058. doi:10.1002/ejoc.202200058

[31] Huang KJ , Pantua H , Diao J , et al. Deletion of a previously uncharacterized lipoprotein lirL confers resistance to an inhibitor of type II signal peptidase in *Acinetobacter baumannii* . Proc Natl Acad Sci USA. 2022;119(38):e2123117119. doi:10.1073/pnas.2123117119 36099298 PMC9499571

[32] Onyedibe KI , Nemeth AM , Dayal N , et al. Re‐sensitization of multidrug‐resistant and colistin‐resistant gram‐negative bacteria to colistin by Povarov/Doebner‐derived compounds. ACS Infect Dis. 2023;9(2):283‐295. doi:10.1021/acsinfecdis.2c00417 36651182 PMC10547215

[33] Goethe O , DiBello M , Herzon SB . Total synthesis of structurally diverse pleuromutilin antibiotics. Nat Chem. 2022;14(11):1270‐1277. doi:10.1038/s41557-022-01027-7 36163267 PMC9633427

[34] Perlmutter SJ , Geddes EJ , Drown BS , Motika SE , Lee MR , Hergenrother PJ . Compound uptake into *E. coli* can be facilitated by N‐alkyl guanidiniums and pyridiniums. ACS Infect Dis. 2021;7(1):162‐173. doi:10.1021/acsinfecdis.0c00715 33228356 PMC7796962

[35] Dean CR , Visalli MA , Projan SJ , Sum PE , Bradford PA . Efflux‐mediated resistance to tigecycline (GAR‐936) in *Pseudomonas aeruginosa* PAO1. Antimicrob Agents Chemother. 2003;47(3):972‐978. doi:10.1128/AAC.47.3.972-978.2003 12604529 PMC149306

[36] Payne DJ , Gwynn MN , Holmes DJ , Pompliano DL . Drugs for bad bugs: confronting the challenges of antibacterial discovery. Nat Rev Drug Discov. 2007;6(1):29‐40. doi:10.1038/nrd2201 17159923

[37] Tommasi R , Brown DG , Walkup GK , Manchester JI , Miller AA . ESKAPEing the labyrinth of antibacterial discovery. Nat Rev Drug Discov. 2015;14(8):529‐542. doi:10.1038/nrd4572 26139286

[38] Martinez‐Fructuoso L , Arends SJR , Freire VF , et al. Screen for new antimicrobial natural products from the NCI program for natural product discovery prefractionated extract library. ACS Infect Dis. 2023;9(6):1245‐1256. doi:10.1021/acsinfecdis.3c00067 37163243 PMC10262198

[39] Richter MF , Hergenrother PJ . Reaction: broad‐spectrum antibiotics, a call for chemists. Chem. 2017;3(1):10‐13. doi:10.1016/j.chempr.2017.06.014

[40] Ling LL , Schneider T , Peoples AJ , et al. A new antibiotic kills pathogens without detectable resistance. Nature. 2015;517(7535):455‐459. doi:10.1038/nature14098 25561178 PMC7414797

[41] Imai Y , Meyer KJ , Iinishi A , et al. A new antibiotic selectively kills gram‐negative pathogens. Nature. 2019;576(7787):459‐464. doi:10.1038/s41586-019-1791-1 31747680 PMC7188312

[42] Zampaloni C , Mattei P , Bleicher K , et al. A novel antibiotic class targeting the lipopolysaccharide transporter. Nature. 2024;625(7995):566‐571. doi:10.1038/s41586-023-06873-0 38172634 PMC10794144

